# Prevalence and socio-economic disparities in vegetarianism and flexitarianism over 15 years: the Dutch Lifelines Cohort

**DOI:** 10.1093/eurpub/ckaf095

**Published:** 2025-06-24

**Authors:** Yinjie Zhu, Marga C Ocké, Emely de Vet

**Affiliations:** Consumption and Healthy Lifestyles Chair Group, Wageningen University & Research, Wageningen, The Netherlands; National Institute for Public Health and the Environment, Bilthoven, The Netherlands; National Institute for Public Health and the Environment, Bilthoven, The Netherlands; Global Nutrition Chair Group, Wageningen University & Research, Wageningen, The Netherlands; Consumption and Healthy Lifestyles Chair Group, Wageningen University & Research, Wageningen, The Netherlands; University College Tilburg, Tilburg School of Humanities and Digital Sciences, Tilburg University, Tilburg, The Netherlands

## Abstract

Transitioning to more plant-based diets is crucial for both planetary and human health, and ensuring an equitable transition across all socio-economic groups is also important. However, empirical evidence on the prevalence and socio-economic disparities in vegetarianism and flexitarianism over time in the same population is scarce. This study investigated this in a general Dutch adult population over 15 years. From three general assessments of the Dutch Lifelines study, 143 359 participants from assessment 1 (2006–2013), 100 859 participants from assessment 2 (2013–2017), and 55 282 participants from assessment 3 (2019–2024) were included in this study. The dietary identity was self-reported, collected at each assessment, and categorized into following a vegetarian, flexitarian, other, and no special diet. Socio-economic status was indicated by education attainment. The association between socio-economic status and different dietary identities was estimated using multinomial logistic regression. The prevalence of individuals following vegetarian or flexitarian diet doubled over the three assessment periods, with the proportion of vegetarians and flexitarians increasing from 2.02% to 4.11% and from 3.50% to 7.16%, respectively. Across three assessments, lower education attainment was consistently associated with a lower likelihood of following a vegetarian or flexitarian diet. For example, in assessment 1 individuals with low education attainment were 77% (relative risk ratio [95% CI]: 0.23 [0.20–0.25]) less likely to follow a vegetarian diet compared to those with high education attainment. In a Dutch population cohort, we observed an increasing trend of vegetarian and flexitarian diets over 15 years, along with persistent socio-economic inequalities in these diets.

## Additional content

The author has now recorded their video: https://oup.cloud.panopto.eu/Panopto/Pages/Viewer.aspx?id=98d2df23-bfea-4c1e-b7ef-b2f000b5ba60.

## Introduction

Shifting diets toward more plant-based foods is essential for reducing the environmental impacts of food production and improving public health. This change supports the “Protein Transition Movement”, which aims to create a sustainable, equitable, and balanced protein system [1]. Embracing plant-based diets aligns with the “EAT-LANCET” commission's recommendations for healthy and sustainable eating, highlighting the dual benefits for both human and planetary health [2, 3]. The transition toward a more plant-based diet at individual level is often executed as vegetarian and flexitarian diets [4, 5]. The consumption of meat and animal-based food products is restricted more in a vegetarian diet compared to a flexitarian diet [6]. The neoteric term “flexitarianism” has gained its popularity in the past decade [7, 8]. Flexitarian often refers to an individual who primarily follow a vegetarian diet but occasionally consume meat. Similar terms in the literature include meat-reducer, meat-avoider, semi-vegetarian, and demi-vegetarian [8].

Understanding how vegetarian and flexitarian diets evolve in a population can provide insightful empirical evidence for stakeholders involved in the protein and sustainability transition. Insights in subgroup differences can help tailor the corresponding interventions and policy measures to ensure an equitable transition, and thus a greater impact. Socio-economic health inequalities have widened over the past decades [9]. Dietary patterns have been shown to be less healthy in individuals with a lower socio-economic position, which could also extend to vegetarian and flexitarian diets [10, 11]. Monitoring these socio-economic differences in vegetarianism and flexitarianism is essential to effectively address and reduce health inequalities. Moreover, socio-economic disparities in vegetarian and flexitarian diets are rarely documented longitudinally within the same population. Acquiring such insights is critical for developing targeted interventions and policies aimed at promoting vegetarian and flexitarian diets across different socio-economic groups.

This study aims to assess the prevalence of and socio-economic disparities in vegetarianism and flexitarianism over a 15-year period using data from a large, general population-based cohort in the Netherlands.

## Methods

### Study population

Lifelines is a multi-disciplinary prospective population-based cohort study examining in a unique three-generation design the health and health-related behaviors of 167 729 persons living in the North of the Netherlands. It employs a broad range of investigative procedures in assessing the biomedical, socio-demographic, behavioral, physical, and psychological factors which contribute to the health and disease of the general population, with a special focus on multi-morbidity and complex genetics. The Lifelines adult study population is representative for the adult population of the north of the Netherlands. A detailed description of the Lifelines study can be found elsewhere [12–14]. Before study entry, a signed informed consent form was obtained from each participant. The Lifelines study was conducted according to the principles of the Declaration of Helsinki and approved by the Medical Ethics Committee of the Institutional Review Board of the University Medical Center Groningen, The Netherlands (2007/152).

Participants recruited at baseline assessment from 2007 to 2013 were invited to participate in the follow-up assessment approximately every 5 years, of which the same set of data and bio samples were also collected. For each follow-up assessments, new participants can also be invited. The current study included adult participants with data on dietary identities at three assessments: the assessment 1 (November 2006–December 2013), assessment 2 (November 2013–December 2017), and assessment 3 (October 2019–January 2024). As a results 143 359, 100 859, and 55 282 participants from assessments 1, 2, and 3, respectively, of which 4.2% and 14.8% participants were newly recruited at assessments 2 and 3, respectively (Supplementary Fig. S1).

### Assessment of dietary identities and socio-demographic determinants

The dietary identity was assessed from multiple self-administered questionnaires. First, participants were asked whether they were following a diet based on certain beliefs/convictions. Subsequently, they were asked which beliefs/convictions they were following with a definition included in the questions, where they could choose from “vegan (no animal products at all)”, “vegetarian (meat less than 1× per week)”, “macrobiotic”, and “anthroposophical”. They were also able to provide qualitative description and comments about their dietary identities if none of the options match their perceived dietary identities. The qualitative description provided by participants were cross-checked with the multiple choices they made by the authors, and then further categorized into individuals who followed vegetarian, flexitarian, other, and no special diet. A vegetarian diet was defined as vegan, vegetarian, pescatarian, macrobiotic, or anthroposophical diets; a flexitarian diet was defined as semi-vegetarian diet, meat-reducers, or self-identified flexitarian in the comments; other diet was defined as a diet according to a specific dietary guidelines, social influencers, nutrients-restricted diet, or any other diets that cannot be considered as vegetarian or flexitarian diets; no special diet was defined when participants indicated that their diets were not based on certain beliefs/convictions and no further comments were given.

Age and sex were recorded at each assessment; SES, as indicated by education attainment, was derived from self-administrated questionnaires at each assessment and further categorized into (1) low—junior general secondary education or lower [International Standard Classification of Education (ISCED) level 0, 1, or 2]; (2) middle—secondary vocational education and senior general secondary education (ISCED level 3 or 4); and (3) high—higher vocational education or university (ISCED level 5 or 6) [15].

### Statistical analyses

The prevalence of types of dietary identities was shown for each assessment cross-sectionally. The socio-demographics were shown for total and across types of dietary identities for each assessment. The associations of age in years, sex, and education attainment and types of dietary identities were investigated using multinomial logistic regression with individuals with high education attainment and no special diet as the reference group. The relative risk ratios (rrrs) with 95% confidence intervals (CIs) as well as the predicted probability of dietary identities across education attainment were presented. Data analyses were performed using R version 4.2.2 (Boston, MA, United States).

## Results

Overall, the prevalence of vegetarianism and flexitarianism doubled over the three assessment periods in a 15-year time span in the whole population, with the proportion of vegetarians and flexitarians increasing from 2.02% to 4.11% and from 3.50% to 7.16%, respectively. The increase of proportion was more prominent from assessment 2 (2013–2017) to 3 (2019–2024), compared to assessment 1 (2006–2013) to 2 (2013-2017). The proportion of individuals following other diets did not change materially cross assessments (Table 1). Across three assessments, the majority of the population with special diets (i.e. either vegetarian, flexitarian, or other diets) were females, and the highest proportion of females was seen in the vegetarian group (Table 1). Among these groups, socio-economic differences were most pronounced in the vegetarian group, followed by the flexitarian and other diet groups (Table 1). Moreover, younger individuals aged <25 and 25–35 years were more likely to follow a vegetarian diet compared to those with no special diet, e.g. 3.8% versus 1.4% and 7.1% versus 3.6%, respectively, in assessment 3. In contrast, older adults aged ≥65 were more likely to follow a flexitarian diet (Table 1).

**Table:** **Table 1.** Participants socio-demographics across three assessments in the Dutch Lifelines cohort

	Assessment 1: November 2006–December 2013					Assessment 2: November 2013–December 2017					Assessment 3: October 2019–January 2024				
	Vegetarian	Flexitarian	Other diet	No special diet	Total	Vegetarian	Flexitarian	Other diet	No special diet	Total	Vegetarian	Flexitarian	Other diet	No special diet	Total
*N*	2903	5012	3500	131 944	143 359	2287	3649	2671	92 252	100 859	2270	3956	1305	47 751	55 282
Prevalence %	2.02	3.50	2.44	92.04	100	2.27	3.62	2.65	91.5	100	4.11	7.16	2.36	86.38	100
Age, years	44 ± 13	48 ± 13	47 ± 12	44 ± 13	44 ± 13	49 ± 13	52 ± 13	49 ± 12	49 ± 13	50 ± 13	53 ± 13	57 ± 12	55 ± 11	56 ± 12	56 ± 12
<25	6.3	3.7	3.2	5.9	5.8	3.9	2.0	2.3	3.2	3.1	3.8	1.8	1.0	1.4	1.6
25–35	18.9	13.0	13.2	16.8	16.6	11.5	9.7	10.7	10.4	10.4	7.1	4.4	2.2	3.6	3.8
35–45	23.8	21.2	26.1	27.8	27.5	18.4	13.8	21.3	19.9	19.7	12.8	9.1	13.1	11.1	11.1
45–55	29.8	30.7	31.1	29.3	29.4	34.7	31.7	36.8	35.1	35.0	21.4	19.5	30.2	24.8	24.4
55–65	15.0	21.5	18.1	13.1	13.6	19.0	23.5	17.6	17.2	17.5	36.6	36.7	34.7	34.4	34.6
≥65	6.2	10.0	8.2	7.0	7.2	12.4	19.2	11.2	14.2	14.3	18.4	28.4	18.8	24.6	24.5
Sex, % male	22.2	25.2	30.9	42.8	41.5	21.1	26.2	29.1	42.4	41	23.2	30.2	28.1	43.0	41.0
Education attainment, %															
Low	14.1	21.5	23.9	29.7	29.0	11.4	17.1	14.7	25.2	24.3	8.2	13.8	12.8	22.7	21.3
Middle	31.3	33.7	36.5	41.1	40.6	29.1	32.3	37.1	41.4	40.7	28.4	32.0	37.8	41.1	39.9
High	54.6	44.9	39.6	29.1	30.4	59.5	50.6	48.2	33.4	35.0	63.3	54.2	49.4	36.2	38.8

**Figure 1.**
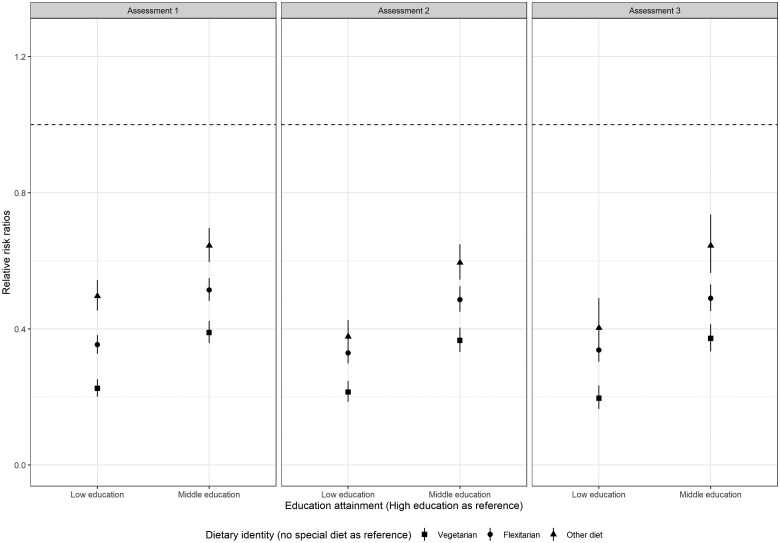
Relative risk ratios from multinomial logistic regressions between education attainment (high education as reference) and different dietary identities (no special diet as reference) at assessments 1, 2, and 3 in the Dutch Lifelines cohort, all models were adjusted for age and sex.

After adjusting for age and sex, compared to individuals with high education attainment, those with lower education attainment were less likely to follow a special diet, with lowest likelihood for vegetarianism, followed by flexitarianism, and other diet (Fig. 1). The relative likelihood across education attainment did not seem to change over time, for instance, compared to individuals with high education attainment, the rrrs [95% CI] of following a vegetarian diet were 0.23 [0.20–0.25] for low education, 0.39 [0.36–0.42] for middle education at assessment 1; 0.22 [0.19–0.25] for low education, 0.37 [0.33–0.40] for middle education at assessment 2; and 0.20 [0.16–0.23] for low education, 0.37 [0.33-0.41] for middle education at assessment 3 (Fig. 1). In each assessment, the probability of individuals with lower education levels following any special diet decreased compared to higher education levels. Conversely, the probability of not following any special diet increased with lower education attainment (Supplementary Fig. S2).

## Discussion

In a general representative Dutch population, the proportion of individuals following a vegetarian and flexitarian diet steadily increased and doubled in the past 15 years. There were always significantly higher proportions of females in the groups with vegetarian, flexitarian, and other diets. In the past 15 years, socio-economic inequalities persisted, with individuals with lower educational attainment being less likely to follow special diets, especially a vegetarian diet.

The most recent prevalence of vegetarians observed is within the range of other reports, including in Germany and New Zealand with 4.11% in our study and others reporting between 4% and 5.4% in the same period [16–18]. The prevalence of vegetarians observed in the earlier period 2013–2017 (2.27%) was lower compared to a reported by the Dutch National Institute for Public Health and the Environment from 2012 to 2016 (4%) [19], but higher compared to that observed in Finland (1.8%) [20]. However, the inconsistent definitions of vegetarian and flexitarian diets currently hinder accurate assessment and comparison across studies and populations [4, 8]. Establishing uniform definitions and tracking trends over time within specific populations are necessary steps to enable reliable analysis of dietary shifts.

A larger discrepancy was observed for flexitarianism with 7.2% in our study compared to previously reported 27%–46% flexitarians from a comparable time period in the Netherlands [21, 22]. Such discrete observations for both vegetarianism and flexitarianism could result from the differences in the study population and the instrument in dietary assessment. First, the Lifelines population was from the northern three provinces of the Netherlands, an area that is less urbanized, and more socio-economically disadvantaged, compared to the rest of the Netherlands [23]. Therefore, we would expect an overall lower prevalence of individuals following special diets. Second, the questionnaire collecting information on special diets from Lifelines was not designed properly for monitoring flexitarian, as flexitarian was not explicitly explained and mentioned as one of the options for special diet [8]. Given that flexitarianism is an emerging new terminology, a certain proportion of flexitarians might consider themselves as with no special diet. Nevertheless, self-defined types of diets were commonly used in literature to identify individuals with a special diet [17, 18, 20].

Our study demonstrates a temporal increase in vegetarianism and flexitarianism within the same population, and most substantially from 2013 to 2024. The considerable increase in vegetarianism and flexitarianism could be allocated to the changes of food environment such as more affordable plant-based meat and dairy alternatives available and more attention from mass media and corporate marketing strategies on climate change and sustainable diets. This could be associated with the launch of EAT-Lancet commission on food, planet, and health in 2019 [3]. Moreover, the positive spillover effects of COVID-19 pandemic cannot be neglected. Studies have shown that COVID-19 pandemic had a great influence on individual’s sustainable consumption and environmental awareness [24, 25], which could lead to a change toward more vegetarianism or flexitarianism [26, 27]. From a local context perspective, the initiation of protein transition movement in academic, policy, and industrial sections in the Netherlands might yield spillover effects in the awareness across difference sections and in the population [1, 28, 29].

Despite the rising trends and popularity of vegetarianism and flexitarianism, the socio-economic inequalities, indicated by education attainment, remained. To achieve an equitable transition toward more sustainable diet at individual level, tailored interventions and policy measures should be allocated to different socio-economically groups to ensure that everyone can contribute to the dietary transition. For instance, dietary counseling had less effects on individuals with lower SES, potentially exacerbating inequalities in dietary identities [30]. Also, policies aimed at promoting vegetarian and flexitarian diets by changing the food environment might face greater resistance among lower educated groups [31]. While educational barriers, such as limited awareness of the health and environmental benefits of vegetarianism and flexitarianism have been noted in lower the SES groups [32], economic concerns also play a significant role. Vegetarian and flexitarian diets are often perceived as more expensive [32], despite evidence suggesting that they can be cost-comparable or even less costly than diets higher in animal-based foods [33]. We speculate that both educational and economic barriers likely contribute to the observed lower adherence to vegetarian and flexitarian diets in lower the SES groups. As such, policy measures should address both information dissemination and affordability to effectively promote dietary shifts across socio-economic strata.

Future research should keep monitoring the population trends and socio-economical inequalities (indicated by multi-faceted indicators) of vegetarian and flexitarian diets with a consensus definition as well as the affordability of these diets. It is equally important to investigate how the trends and transition of vegetarianism and flexitarianism contribute to the intake of animal-based and plant-based foods, and nutritional adequacy and status of the general population and specific subgroups [34]. Furthermore, research on the impact of different dietary consumption on environmental and health outcomes and how socio-economic differences in the adoption of vegetarian and flexitarian diets contribute to health inequalities is warranted to ensure an equitable healthy protein transition [28]. Additionally, the higher likelihood of younger individuals (<25 and 25–35 years) following a vegetarian diet, particularly in the most recent assessment 3, may reflect age-related or generational differences in dietary preferences [35]. The widening discrepancy between the prevalence of vegetarian and no special diets among younger groups over time suggests a possible shift in norms, attitudes, or values toward plant-based eating among newer generations [36]. However, future research is needed to investigate whether these patterns represent a broader generational trend or are shaped by other contextual factors.

To our knowledge, this is the first study reporting the observed trends in vegetarianism and flexitarianism and its socio-economic inequalities in the same larger population over time in the Netherlands. However, a key limitation is the definition of “vegetarian” as consuming meat less than once per week, which may include individuals better described as flexitarians. This could lead to an overestimation of vegetarian prevalence; however, the consistent use of this definition across all assessments supports the validity of internal comparisons. Additionally, the self-reported nature of dietary identity is subject to reporting bias. However, we were able to validate it against dietary intake data from the food frequency questionnaire at assessment 1. At this time point, median (IQR) meat intake followed a clear and expected gradient across dietary identity groups: 0 (0–0) g/d for vegetarians, 35.2 (15.0–61.5) g/d for flexitarians, 65.6 (38.0–89.7) g/d for other diets, and 78.2 (62.0–100.8) g/d for those reporting no special diet (Supplementary Table S1). These patterns support the internal validity of the self-reported categories. Nevertheless, similar cross-checks were not possible at assessments 2 and 3 due to the lack of comparable dietary intake data, limiting our ability to assess the consistency of dietary identity reporting over time. Despite this, the strong alignment at baseline provides some reassurance regarding the accuracy of participants’ self-reporting. Moreover, the Lifelines cohort is representative of adults in the north of the Netherlands; however, regional differences in socio-economic status (SES) and dietary behaviors may limit the generalizability of these findings to other populations. Lastly, two types of selection bias could occur. Individuals identifying as vegetarians or flexitarians were slightly more likely to remain in the study across assessments, particularly from assessment 2 to 3 (e.g. 2.5% vegetarians and 4.0% flexitarians among those followed up vs. 2.1% and 3.3% among those lost to follow-up) (Supplementary Table S2). This may have led to a modest overestimation of their prevalence in assessments 2 and 3. We also acknowledge that individuals with a low education level were more likely to be lost to follow-up (Supplementary Table S2), this may have biased our results toward the null, potentially underestimating the strength of the association between education and likelihood of following a vegetarian or flexitarian diet. Thus, we would expect that the true SES disparity in dietary identities be greater than observed in the study.

In conclusion, we observed a rising trends of vegetarian and flexitarian diets in a general Dutch population and persistent socio-economic inequalities in vegetarianism and flexitarianism over 15 years.

## Supplementary Material

ckaf095_Supplementary_Data

## Data Availability

Data described in the article, codebook, and analytic code will not be made available because the authors do not have the authority to share them according to Lifelines data access permissions. But any researchers can apply to use Lifelines data, including the variables used in this investigation. Information about access to Lifelines data is given on their website: https://www.lifelines-biobank.com/researchers/working-with-us/step-1-prepare-and-submit-your-application.
