# Supplementary material for: Prevalence and socio-economic disparities in vegetarianism and flexitarianism over 15 years: the Dutch Lifelines Cohort
Source: Eur J Public Health. 2025 Jun 24;35(4):714–9. doi: 10.1093/eurpub/ckaf095 (PMC12311360; doi:10.1093/eurpub/ckaf095)
Supplement: ckaf095_Supplementary_Data [file ckaf095_supplementary_data.docx]

**Supplementary Information**

**
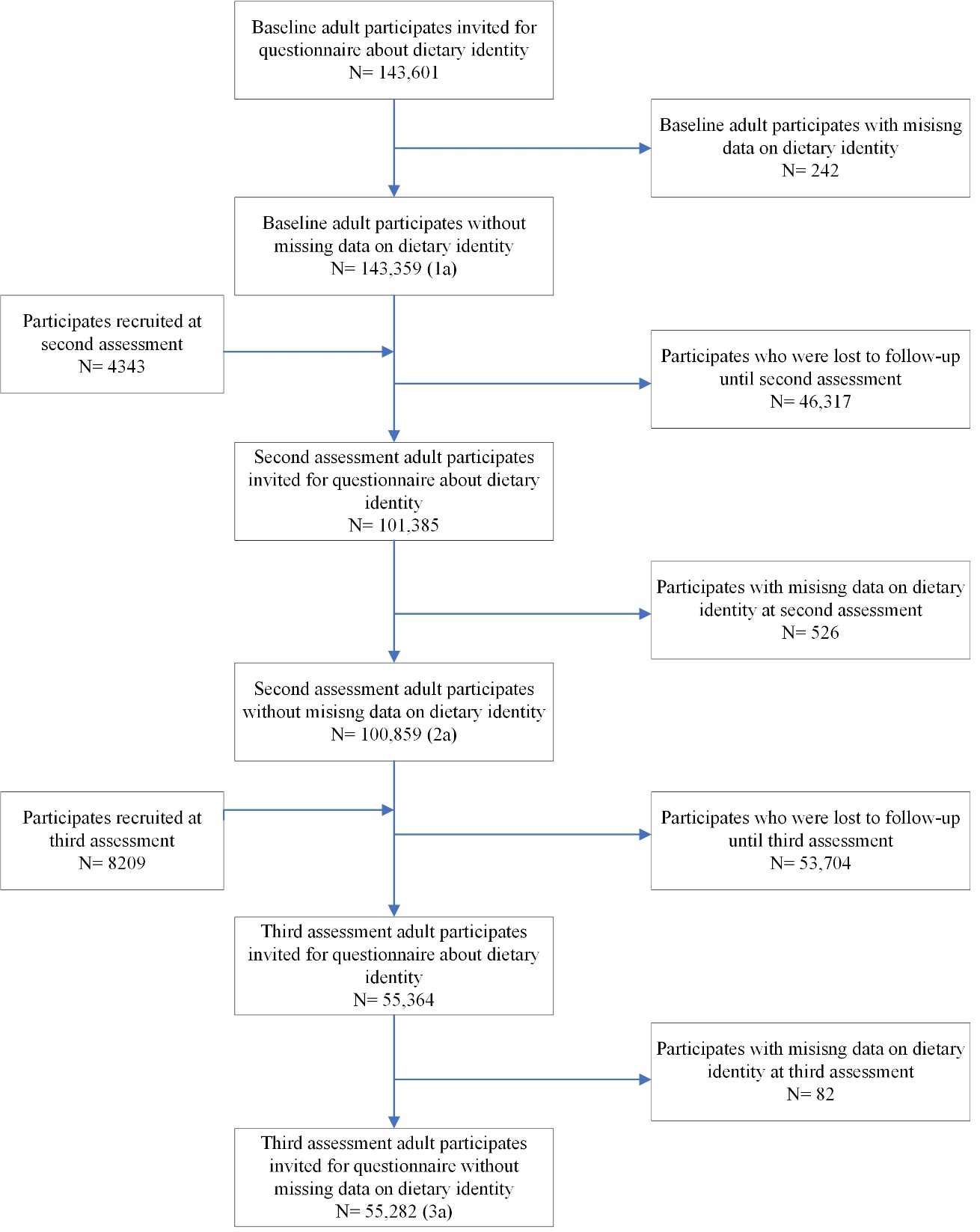
**

**Supplementary Figure S1. Lifelines Study population selection at three assessments.**


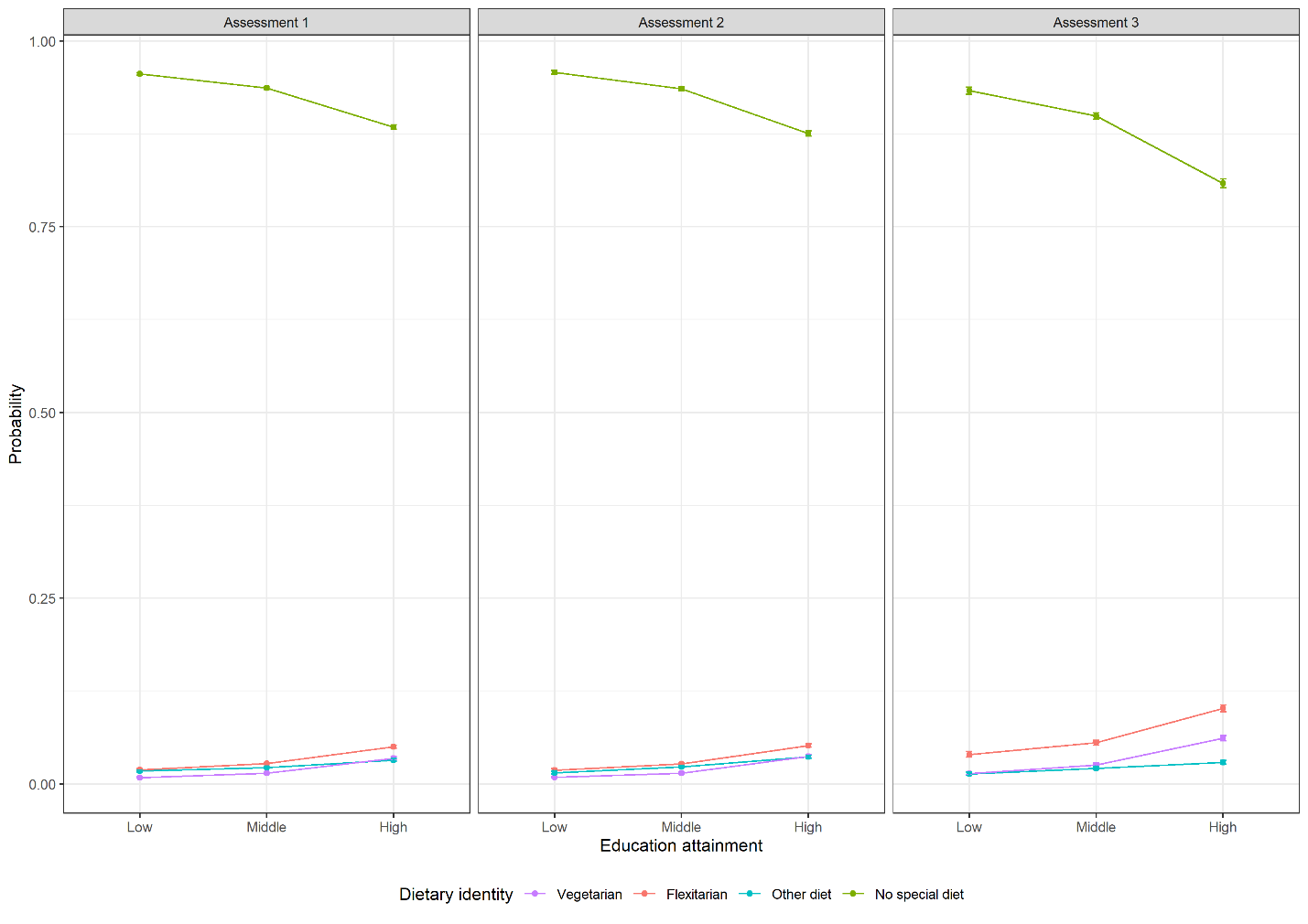


**Supplementary Figure S2. Probability of dietary identities across education attainment at assessment 1, 2, and 3 in the Dutch Lifelines cohort, all models were adjusted for age and sex.**

**Supplementary Table S1. Meat intake in mean (SD) and animal protein intake in median (IQR) among dietary identities groups at assessment 1.**

|  | Vegetarian | Flexitarian | Other diet | No special diet |
| --- | --- | --- | --- | --- |
| Meat intake, g/day | 0 (0-0) | 35.2 (15.0-61.5) | 65.6 (38.0-89.7) | 78.2 (62.0-100.8) |
| Animal protein, g/day | 27.6 ± 12.3 | 36.4 ± 13.6 | 42.5 ± 16.0 | 44.6 ± 13.9 |

*Meat intake and animal protein intake was assessed by a food frequency questionnaire and was in line with the meat category from the lifelines diet score ^1^

**Supplementary Table S2. Dietary identities education attainment of individuals lost-to follow up throughout the assessments.**

|  | Assessment 1 (n=143,359) | | Assessment 2 (n=100,859) | |
| --- | --- | --- | --- | --- |
|  | Lost to follow-up at assessment 2 (n=46,317) | Follow-up at assessment 2 (n= 97,042) | Lost to follow-up at assessment 3 (n=53,704) | Follow-up at assessment 3 (n= 47,155) |
| Dietary identity, % |  |  |  |  |
| Vegetarian | 2.0 | 2.1 | 2.1 | 2.5 |
| Flexitarian | 3.2 | 3.6 | 3.3 | 4.0 |
| Other diet | 2.4 | 2.4 | 2.6 | 2.6 |
| No special diet | 92.4 | 91.9 | 92.0 | 90.9 |
| Education attainment, % |  |  |  |  |
| Low | 30.0 | 28.5 | 31.5 | 24.4 |
| Middle | 41.9 | 39.9 | 35.3 | 34.7 |
| High | 28.1 | 31.6 | 33.2 | 40.9 |

**Reference**

1 Vinke PC, Corpeleijn E, Dekker LH, Jacobs DR, Jr., Navis G, Kromhout D. Development of the food-based Lifelines Diet Score (LLDS) and its application in 129,369 Lifelines participants. Eur J Clin Nutr 2018;72:1111-1119.
